# Parental Factors Associated with Obesity in Korean Adolescents

**DOI:** 10.3390/ijerph17145126

**Published:** 2020-07-16

**Authors:** Heun Keung Yoon, Gwang Suk Kim, Suhee Kim

**Affiliations:** 1 Department of Nursing, Wonju College of Medicine, Yonsei University, Gangwon-do 26493, Korea; yhk9874@hanmail.net; 2 Mo-Im Kim Nursing Research Institute, College of Nursing, Yonsei University, Seoul 03722, Korea; gskim@yuhs.ac; 3 School of Nursing and Research Institute of Nursing Science, Hallym University, Gangwon-do 24252, Korea

**Keywords:** adolescent, obesity, parents, perception, lifestyle

## Abstract

Parental characteristics can influence adolescent obesity. However, the influence of parental characteristics on obesity may differ depending on the adolescent’s sex. This study evaluated parental characteristics that were associated with obesity in male and female adolescents. This study involved the secondary data analysis of cross-sectional survey data that were collected from June to September 2015. The study subjects included 1621 eighth-grade students. The study variables included sex, age, body mass index, household income, parental weight, parental perceptions of the child’s body, parental lifestyle, and parental social support for healthy eating and physical activity. The association between parental factors and adolescent obesity was analyzed via logistic regression analysis for each sex. Among male students, the fathers’ and mothers’ overweight status, fathers’ underestimation and overestimation of male adolescent weight, mothers’ dietary habits, and the mothers’ physical activity level were identified as obesity-associated factors. Among female students, the mothers’ overweight status, underestimation of female adolescent weight by fathers, dietary habits, and the physical activity level of fathers were significantly associated with adolescent obesity. The mothers’ overweight status and the underestimation of weight by fathers were strongly associated with obesity in male and female adolescents. Parental involvement in obesity-intervention programs could help prevent adolescent obesity.

## 1. Introduction

Over the past decade, obesity rates have steadily increased among children and adolescents across Korea [1]. According to the results of a national health examination of students that was conducted in 2018, the obesity rates, including overweight, were 26.8% (male) and 21.1% (female) among elementary schoolers, 26.7% (male) and 22.4% (female) among middle schoolers, and 29.1% (male) and 25.1% (female) among high schoolers [2]. A comparison of these rates with those that were measured a decade earlier indicated the greatest obesity increase in middle schoolers among the school-aged children and adolescents [1].

Adolescent obesity is associated with increased risks of adult diabetes, stroke, cardiovascular disease and hypertension, as well as cancer, and consequently increases the risk of early mortality [3]. Moreover, adolescent obesity leads to psychosocial problems, such as a negative self-image, low self-esteem, depression and anxiety [4]. Therefore, obesity prevention is important for ensuring an adolescent’s physical and mental health.

Several factors are related to adolescent obesity. A systematic review [5] reported that high calorie intake and excessive fast food intake were associated with high body mass index (BMI). Furthermore, the lack of physical activity and sleep time were related to high BMI and the presence of specific genes in obesity. In addition, the community environment has been reported to affect youth obesity. For example, studies have shown that high green space availability and large public sports facilities are associated with less obesity [6,7].

Particularly, previous studies have shown the associations of parental characteristics with overweight and obesity in adolescents. With regard to socioeconomic factors, a low household income increases the possibility of adolescent obesity [8], and many studies have suggested that parental weight is a strong contributory factor to increased childhood obesity [9,10,11]. In addition, most parents of overweight children tend to underestimate their children’s weight status; therefore, parental perceptions of their child’s weight play a critical role in obesity prevention [12,13,14]. An adolescent’s obesogenic dietary habits and physical activities are affected by those of their family [15,16]. A recent study of parental modeling of healthy dietary behavior and frequent physical activity predicted an associated reduction in BMI among overweight and obese adolescents [17].

A review found that child and parental dietary intakes varied among parent–child pairs [18]. The parental influence on adolescents depends not only on the sexes of the parents and children, but also on societal and cultural factors and values. Korea has traditionally been a patriarchal society, although recent changes in social phenomena have considerably changed this societal structure [19,20]. Patriarchy is a social system wherein men hold the primary roles of power and predominance in political leadership, moral authority, social privilege and property control [20]. Thus, the identification of the distinct influences of fathers and mothers in this changing society will facilitate the development of practical intervention programs for the prevention of adolescent obesity. Therefore, this study aimed to investigate the paternal and maternal characteristics associated with obesity in male and female adolescents.

## 2. Materials and Methods

### 2.1. Study Design and Participants

This secondary data analysis was based on existing cross-sectional survey data (source deleted for blinded review). The institutional review board (IRB) approved the methodology of this study (IRB approval no. 2017-0002).

The primary survey data were collected from June to September 2015, and were used to identify the various factors associated with youth obesity. The primary study participants were eighth-grade students at middle schools in Seoul. By using a convenience sampling method, 50 of 383 middle schools in Seoul were selected. The same method was then used to select classrooms from each school. The researcher or research assistants who underwent training for this study explained the purpose of the study during the homeroom periods, and conveyed the voluntary nature of participation. Students in the selected classes received a document that explained the study details (intended for the students and their guardians), a written consent form (for the student and their guardians), a questionnaire, and a vitamin C supplement worth USD 2 as compensation. Students were asked to complete and return the questionnaire and written consent form within 1 week.

The following self-reported eligibility criteria were applied to the primary study: (1) agreement to participate in the research and ability to convey information; (2) no existing diseases that could influence weight status, including thyroid dysfunction, diabetes mellitus, heart disease and growth hormone deficiency; and (3) no increase in muscle mass, as would occur in athletes. The exclusion criteria in this study were specified to explore the relationship between parental characteristics and the prevalence of childhood obesity: underweight students were excluded to limit the comparison to normal-weight and obese students, and cases lacking data on parental characteristics, such as household income, parental weight status and parental perceptions, were excluded.

This survey was conducted among 2766 eighth-grade students (with 101 classrooms in 50 schools); however, 2617 questionnaires were returned (response rate, 94.6%). Among the excluded subjects, 25 were excluded for refusal of consent for study participation, 46 for diseases that may affect their weight status, 49 as they were athletes, 333 because of missing data on the main variables (height and weight), and 95 as they were underweight. Additionally, 440 subjects were excluded because of missing data on parental variables. The target age range in this study was 13–14 years; therefore, eight subjects were excluded because they were 12 or 15 years old. Finally, 1621 adolescents were included in this study.

### 2.2. Measurement

The primary survey comprised approximately 100 questions. In this study, BMI was used as a dependent variable, and parental characteristics (household income, parental weight, parental perceptions of the child’s body, parental lifestyle, and parental social support for healthy eating and physical activity) were included as independent variables. All answers were self-reported by the adolescents.

#### 2.2.1. Adolescent BMI

The height and weight measurements of middle school students were self-reported. Students were stratified by their BMI based on the 2007 Korean National Growth Chart, which was developed using Korean child–adolescent data in accordance with the United States Centers for Disease Control and Prevention methodology [21]. Obesity was defined as a BMI at or above the 95th percentile, and overweight was defined as a BMI at or above the 85th percentile and below the 95th percentile for Korean adolescents of the same age and sex [21]. Regardless of the percentile, obesity was determined using a BMI cutoff of 25 kg/m^2^. Normal weight was defined as a BMI at or above the 5th percentile, and below the 85th percentile [21]. In this study, overweight students were categorized into the “obesity” group to enable a comparison between the normal-weight and overweight/obesity subgroups.

#### 2.2.2. Household Income

One survey item addressed household income, and the result was self-reported by the adolescents. The question was, “What is the average monthly income of the family, including both the father’s and mother’s incomes?” Five response options were provided: very low, low, moderate, high or very high.

#### 2.2.3. Child’s Perception of Parents’ Weight Status

Adolescents were asked to respond to two questions concerning their parents’ body shapes. “How would you describe your father’s body shape?” and “How would you describe your mother’s body shape?” The adolescent was asked to select one of five response options: underweight, normal, overweight, obese or other.

#### 2.2.4. Child’s Perception of Parental Perception of the Adolescent’s Body Shape

Adolescents were asked to respond to two questions on their parents’ perceptions of the adolescent’s body: “What does your father think about your body shape?” and “What does your mother think about your body shape?” The adolescent was asked to respond with one of the following five options: underweight, normal, overweight, obese or other. Subsequently, the adolescent’s answers to these questions were compared with his/her actual BMI percentile to determine the parental perception of the adolescent’s body, which was classified as “underestimated,” “accurate,” or “overestimated.”

#### 2.2.5. Child’s Perception of Parental Healthy Dietary Habits and Physical Activity

Adolescents were asked to respond to one question on parental healthy dietary habits: “What is your father’s/mother’s usual healthy eating score on a 10-point scale?” The questionnaire clarified “healthy eating” as the less frequent consumption of unhealthy foods, such as fatty foods and soft drinks, and more frequent consumption of healthy foods, such as vegetables and water. Adolescents used the numeric rating (10-point scale bar) to assign higher scores to parents with healthy dietary habits; therefore, higher scores indicated a subjective assessment of higher levels of healthy eating.

Similarly, adolescents were asked to respond to one question on the parental levels of physical activity: “What is your father’s/mother’s usual physical activity score on a 10-point scale?” The adolescents specified a value by using a numeric rating (10-point scale bar). The relevant physical activities included activities such as walking, housework and dancing, as well as sports and all forms of aerobic activities that require the use of muscles and bones. Higher scores indicated a subjective assessment of higher levels of physical activity.

#### 2.2.6. Perceived Social Support for Healthy Eating

The adolescents’ perceived measures of social support for healthy eating (SSHE) were based on a modified version of the method developed by Stanton and colleagues [15] that was adapted by Fitzgerald and colleagues [22]. Because no Korean translation was available, the metric was first translated from English into Korean and then reviewed by three experts before use. Four items were rated on a 5-point scale, ranging from “1, not at all” to “5, strongly agree.” A higher score indicated a higher level of SSHE. Fitzgerald reported a Cronbach’s α of 0.73 for this metric [22]. In this study, the corresponding Cronbach’s α value was 0.892.

#### 2.2.7. Perceived Social Support for Physical Activity

The adolescents’ perceived measures of social support for physical activity (SSPA) were based on a modified version of the Social Support and Exercise Survey instrument, developed by Sallis and colleagues [23] and later adapted by Roh and colleagues [24]. Six items were rated on a 5-point scale from “1, not at all” to “5, strongly agree.” A higher score indicated a higher level of SSPA. Sallis [23] and Roh [24] reported Cronbach’s α values of 0.91 and 0.93, respectively, for this scale [23,24]. In this study, the corresponding Cronbach’s α value was 0.922.

### 2.3. Data Analysis

Descriptive statistics (means, standard deviations and proportions) were determined for all variables. Differences between the adolescent and parental characteristics (adolescent sex and age, household income, parental weight statuses, parental perceptions of the child’s body, parental healthy dietary habits and physical activity, and parental support) as concerns the normal-weight and overweight/obese groups were analyzed by using the chi-squared test and the Student’s *t*-test (two-tailed). After univariate analysis, the parental characteristics were subjected to sex-stratified logistic regression analysis to identify the parental factors that were associated with adolescent obesity. Cronbach’s α was used to identify the internal consistency and reliability of the SSHE and SSPA. All analyses were conducted using IBM SPSS version 23 (SPSS Inc., Chicago, IL, USA), and the significance level was set at *p* < 0.05.

## 3. Results

A slightly larger proportion of male adolescents than female adolescents were classified as overweight/obese (14.7% vs. 11.8%), although this difference was not significant. An analysis of the overweight/obese status according to parental characteristics revealed statistically significant differences in the fathers’ and mothers’ weight statuses (*p* = 0.016 and < 0.001, respectively), and in the fathers’ and mothers’ perceptions of the child’s body (*p* < 0.001 for both) (Table 1).

A logistic regression analysis stratified by sex was applied to identify parental factors that might explain the participants’ overweight/obese status (Table 2). Among male students, the father’s overweight status [odds ratio (OR) = 1.761, *p* = 0.031], mother’s overweight status (OR = 1.710, *p* = 0.040), father’s underestimation of a child’s weight (OR = 3.271, *p* < 0.001), father’s overestimation of a child’s weight (OR = 0.250, *p* = 0.021), mother’s healthy dietary habits (OR = 0.849, *p* = 0.035), and mothers’ level of physical activity (OR = 1.116, *p* = 0.039) were significantly associated with adolescent obesity. Among female students, the mother’s overweight status (OR = 2.082, *p* = 0.003), father’s underestimation of a child’s weight (OR = 2.031, *p* = 0.018), father’s healthy dietary habits (OR = 0.810, *p* = 0.001) and father’s level of physical activity (OR = 1.156, *p* = 0.010) were significantly associated with adolescent obesity.

## 4. Discussion

The male and female adolescents included in this study had overweight/obesity rates of 14.7% and 11.8%, respectively. These rates were consistent with the figures published by the Korea Youth Risk Behavior Web-based Survey—a self-reported national youth survey conducted in 2012—which yielded overweight/obesity rates of 14.1% and 12.0% for male and female youths, respectively [25]. Despite these similar findings, the overweight/obesity rate was lower than the actual rate, due to self-reporting (male middle schoolers, 26.7%; female middle schoolers, 22.4%) [2]. In the United States, the reported prevalence of adolescent obesity among respondents in the age range of 12–19 years was 20.6% [26], which was relatively higher than the Korean adolescent obesity rate (male middle schoolers, 16.0%; female middle schoolers, 12.4%) [2]. Nonetheless, the obesity rates of Korean adolescents have increased during the last 10 years [1]. Therefore, adolescent obesity should be managed as a major healthcare issue.

This study identified the parental characteristics associated with obesity in male and female adolescents. These parental characteristics are discussed below.

### 4.1. Parental Weight Status

In this study, mothers’ overweight/obese status was associated with obesity in both male and female adolescents, whereas the fathers’ overweight/obese status was only associated with obesity in male adolescents. Many previous studies have indicated that the likelihood of obesity in both male and female adolescents increases if the mother is overweight/obese [10,11,27]. In contrast, relatively few studies have focused on the father’s influence on obesity in their children, and the reported findings with regard to the relationship between a father’s weight and childhood obesity by sex have been inconsistent. In one study, the father’s obesity was found to impact both male and female offspring [10]; however, another study reported that a father’s BMI was correlated with his daughter’s weight and not his son’s weight [28]. A Chinese study found that both mothers and fathers had an impact on their children’s BMI, although mothers had a relatively higher impact [29]. Although adolescent obesity may be due to genetic traits, interventions for adolescent obesity should allow the co-participation of mothers and fathers, as parental obesity-related behaviors may affect children.

### 4.2. Parents’ Perception of a Child’s Body

This study found that fathers and mothers underestimated their children’s weight in 26.2% and 26.7% of cases, respectively. A previous meta-analysis found that half of parents underestimated the overweight status of their child [13]; however, little is known with regard to the reasons for this phenomenon. A study reported that parents who preferred a heavier child underestimated their child’s weight more than parents who preferred a leaner child [30]. In addition, mothers underestimate their child’s weight because they believe that overweight in childhood is an indicator of a good nutritional status, and that a child will eventually increase in both height and weight with normal growth [31]. Accordingly, an intervention program should include content to assist parents in accurate self-assessment and in the assessment of their child’s body weight. Moreover, parents should be advised on how to judge a healthy body via factors that are not based on visual appearance. For example, it is necessary to educate the parents with regard to the amount of muscle and the amount of fat, regardless of the body weight, depending on the child’s physical condition, as these factors may affect growth and development in the future.

This study indicated that the likelihood of obesity in male and female adolescents increased when the father underestimated the actual weight of the adolescent; however, a mother’s underestimation of her child’s weight did not affect the likelihood of adolescent obesity in either sex. This result was consistent with that of previous research, which showed that parental misperceptions affect childhood obesity [14]. However, the fact that only the perception of the father, not the mother, has an influence on a child’s weight is a unique result. The effect of a father’s perception may be attributed to the patriarchal social characteristics in Korea. In a patriarchal society, the man is traditionally regarded as the head of household, and has authority over the general proceedings of the house. Therefore, a father’s direct or indirect perceptions and expressions about a child’s weight may affect the child’s thoughts and emotions. For example, if a child is actually obese and the authoritative father recognizes it as healthy, then it is likely that the child will also think so. However, if the father thinks his child is obese, regardless of the actual situation, his child might think this as well, and try to lose weight. In comparison, the influence of a mother in a patriarchal society is relatively weak. Mothers are known to play a crucial role in childhood obesity through the preparation of food at home, which therefore influences a child’s eating habits [28,32]. Thus, a father’s accurate perception of childhood weight, and fathers’ positive attitudes toward adolescent obesity management, can further maximize the effectiveness of obesity-prevention programs.

### 4.3. Parents as Role Models: Dietary Habits and Physical Activity

This study found that when a mother had healthy dietary habits, her son faced a decreased risk of obesity, consistent with the results of previous research, wherein the parental modeling of healthy dietary habits affected the child’s dietary habits and reduced the child’s BMI [17]. As mentioned, mothers are primarily in charge of providing food for children at home [28,32]; therefore, it stands to reason that a mother’s dietary habits would have a major impact on her child’s eating environment. This concept is supported by a study that reported a significant relationship between the dietary intakes of mothers and sons, including carbohydrates, proteins, fats, saturated fats and cholesterol [28].

In the results of this study, the fathers’ healthy dietary habits were associated with female adolescent obesity. A previous study found sex-specific associations between parenting practices—including modeling and encouraging healthy eating and physical activity—and adolescent BMI among opposite-sex parent-adolescent dyads (i.e., mother-son and father-daughter) [33]. Furthermore, the effects of these opposite-sex parent–adolescent dyads are supported by a study, which reported that children within opposite-sex dyads exhibited greater weight loss than children in same-sex dyads within a family-based obesity treatment program [34]. Therefore, the fact that the dietary habits of opposite-sex parents, rather than those of same-sex parents, play an important role in adolescent obesity should be considered when planning adolescent obesity-management programs.

Unlike dietary habits, when the physical activity levels of the mother increased, the risk of obesity in her son increased. A similar relationship was observed for father–daughter dyads. Although many reports demonstrated that the perceived modeling of parental physical activity had a positive impact on a child’s participation in physical activities [35,36], a systematic review of 14 studies revealed no correlation (seven studies, 50.0%), or even a negative relationship (one study, 7.1%), between the physical activity levels of a child and his or her parents [37]. In Korea, 47.0% of adults and 78.0% of adolescents do not achieve the recommended levels of physical activity [38]. The physical activity rate is very low in practice, and if a family member becomes obese, regular exercise begins in response to this weight status. Therefore, the relationship between parental physical activity levels and adolescent obesity should be interpreted cautiously in cross-sectional studies, such as the present research. Further investigations, including longitudinal studies at the national level, may help to more clearly identify the relationship between adolescent obesity and parental physical activity.

### 4.4. Social Support for Healthy Eating and Physical Activity

In this study, social support for healthy eating and physical activity was not associated with obesity in either male or female adolescents. In another recent study, parental behaviors were reported to have a larger impact on children than parental verbal motivation [17]. Moreover, these findings support the conclusion that a parent’s actual behavior with regard to healthy eating habits is more effective in preventing adolescent obesity than their making recommendations to eat healthy food.

### 4.5. Limitations

This study has several limitations. First, our reliance on a self-reported questionnaire may have led to the underestimation of the BMI status of adolescents, and an overestimation of household income. In addition, because the contents of the parents’ characteristics were investigated by the children and the measurement tools were not previously verified, there might have been differences between the adolescents’ perception of parental characteristics (e.g., weight, perceptions of a child’s body shape, dietary habits and levels of physical activity) and the actual parental characteristics. In particular, the variables pertaining to the parental perception of a child’s body shape (i.e., underestimation, accurate perception, overestimation of a child’s weight status) were ascertained from the self-reported BMI and the children’s perception of parental perception of a child’s body shape; therefore, it might have resulted in a double burden of underestimation or overestimation. Thus, the results of this study should be interpreted with this limitation in mind. Third, in this study, adolescent obesity was classified based on the Korean National Growth Chart. This may limit the comparison of the rates identified in this study with the obesity rates in different countries. Fourth, a cross-sectional research design was used to analyze the data, which made it difficult to infer causal associations. Therefore, the relationships between parental physical activity levels and adolescent obesity should be interpreted cautiously. Finally, as a secondary analysis, this study did not include various variables such as genetic and environmental variables. In addition, this study did not include variables such as the adolescents’ perceptions of their own body weight or the levels of parental social support stratified by sex. Thus, the causal effects of parental characteristics on adolescent obesity were not identified.

## 5. Conclusions

Despite these limitations, this study identified parental weight, parental perceptions of a child’s weight and parental dietary habits as factors affecting the prevalence of adolescent obesity. In the patriarchal society of Korea, a father’s perceptions may have a more significant influence on adolescents than a mother’s perceptions. In addition, adolescents were more affected by the health behaviors of the parent of the opposite sex than by those of the parent of the same sex. These results provide basic data to support the development of effective obesity-management programs for adolescents. Based on the abovementioned findings, we suggest the following: first, efforts are needed to ensure that parents form accurate perceptions of their child’s body, which would facilitate the effective prevention of adolescent obesity. To do this, parents should have more specific knowledge on the normal ranges of height, weight, amount of muscle and amount of fat, rather than reliance on the visible body form. Second, we suggest that parents serve as role models by eating healthy foods, rather than by merely talking about the benefits of a healthy diet. In particular, the finding that adolescents are affected by the habits or behaviors of the parent of the opposite sex should be considered when developing effective obesity-management programs for adolescents. Finally, in Korea, where patriarchal thinking is predominant, the attitudes and evaluations of a father have a great influence on the children; therefore, not only the mothers, who are in charge of meals at home, but also the fathers should participate directly or indirectly in obesity-management programs.

## Figures and Tables

**Table 1:** Overweight/obese status according to adolescent and parental characteristics (*n* = 1621).

Characteristics *n* (%) or Mean ± SD	Total	Normal Weight	Overweight/Obese	X^2^/t (*p*)
	*n* = 1621 (100)	*n* = 1409 (86.9)	*n* = 212 (13.1)	
**Adolescent characteristics**				
Sex				
Male	712 (43.9)	607 (85.3)	105 (14.7)	3.111 (0.078)
Female	909 (56.1)	802 (88.2)	107 (11.8)	
Age	13.92 ± 0.31	13.92 ± 0.31	13.88 ± 0.31	1.960 (0.050)
**Parental characteristics**				
Household income				
Low	114 (7.0)	93 (81.6)	21 (18.4)	3.079 (0.214)
Middle	1026 (63.3)	896 (87.3)	130 (12.7)	
High	481 (29.7)	420 (87.3)	61 (12.7)	
Father’s weight status				
Underweight	198 (12.2)	177 (89.4)	21 (10.6)	8.314 (0.016)
Normal	918 (56.6)	811 (88.3)	107 (11.7)	
Overweight	505 (31.2)	421 (83.4)	84 (16.6)	
Mother’s weight status				
Underweight	231 (14.3)	210 (90.9)	21 (9.1)	21.587 (<0.001)
Normal	977 (60.3)	867 (88.7)	110 (11.3)	
Overweight	413 (25.4)	332 (80.4)	81 (19.6)	
Father’s perception of child’s body				
Accurate	924 (57.0)	829 (89.7)	95 (10.3)	47.436 (<0.001)
Underestimated	424 (26.2)	328 (77.4)	96 (22.6)	
Overestimated	273 (16.8)	252 (92.3)	21 (7.7)	
Mother’s perception of child’s body				
Accurate	912 (56.3)	811 (88.9)	101 (11.1)	23.084 (<0.001)
Underestimated	432 (26.7)	347 (80.3)	85 (19.5)	
Overestimated	277 (17.1)	251 (90.6)	26 (9.4)	
Father’s healthy dietary habits	7.00 ± 2.30	7.03 ± 2.29	6.80 ± 2.37	1.393 (0.164)
Mother’s healthy dietary habits	7.62 ± 2.01	7.65 ± 1.98	7.40 ± 2.14	1.697 (0.090)
Father’s level of physical activity	6.88 ± 2.46	6.86 ± 2.46	7.07 ± 2.51	−1.157 (0.248)
Mother’s level of physical activity	6.41 ± 2.30	6.40 ± 2.29	6.52 ± 2.35	−0.721 (0.471)
SSHE ^a^	3.49 ± 0.96	3.50 ± 0.96	3.47 ± 0.97	0.373 (0.709)
SSPA ^b^	3.05 ± 0.99	3.04 ± 0.99	3.11 ± 0.97	−0.938 (0.348)

^a^ SSHE = social support for healthy eating; ^b^ SSPA = social support for physical activity.

**Table 2:** Logistic regression model for overweight/obese status among adolescents by sex.

Characteristics (Reference)	Overweight/Obese	
	Male OR (95% CI)	Female OR (95% CI)
Father’s weight status (normal)		
Underweight	0.412 (0.170–1.001)	1.029 (0.529–2.000)
Overweight	1.761 * (1.054–2.941)	1.481 (0.905–2.422)
Mother’s weight status (normal)		
Underweight	1.085 (0.528–2.227)	0.547 (0.259–1.156)
Overweight	1.710 * (1.024–2.857)	2.082 ** (1.290–3.362)
Father’s perception (accurate)		
Underestimated	3.271 *** (1.715–6.238)	2.031 * (1.129–3.652)
Overestimated	0.250 * (0.077–0.814)	0.772 (0.396–1.505)
Mother’s perception (accurate)		
Underestimated	1.205 (0.630–2.303)	0.922 (0.501–1.697)
Overestimated	1.061 (0.371–3.036)	0.735 (0.395–1.370)
Father’s healthy dietary habits	1.124 (0.978–1.290)	0.810 ** (0.718–0.913)
Mother’s healthy dietary habits	0.849 * (0.729–0.989)	0.996 (0.874–1.135)
Father’s level of physical activity	1.018 (0.915–1.133)	1.156 * (1.036–1.291)
Mother’s level of physical activity	1.116 * (1.006–1.238)	1.016 (0.910–1.135)
SSHE ^a^	0.963 (0.731–1.270)	0.999 (0.754–1.324)
SSPA ^b^	0.930 (0.711–1.216)	1.281 (0.976–1.681)

Results are adjusted for adolescents’ age and household income. ^a^ SSHE = social support for healthy eating; ^b^ SSPA = social support for physical activity; * *p* < 0.05; ** *p* < 0.01; *** *p* < 0.001.

## References

[1] Kwon E., Nah E.-H. (2016). Secular trends in height, weight and obesity among Korean children and adolescents in 2006–2015. Korean J. Health Educ. Promot..

[2] Statistics Korea, Ministry of Gender Equality & Family 2019 Youth Statistics Report. http://www.mogef.go.kr/nw/rpd/nw_rpd_s001d.do?mid=news405&bbtSn=706338.

[3] Xu S., Xue Y. (2015). Pediatric obesity: Causes, symptoms, prevention and treatment. Exp. Ther. Med..

[4] Kang N.R., Lee J.S., Kang K.S., Kwack Y.S. (2016). Mental Health Problems in Child and Adolescent Obesity. J. Korean Acad. Child Adolesc. Psychiatry.

[5] Narciso J., Silva A.J., Rodrigues V., Monteiro M.J., Almeida A., Saavedra R., Costa A.M. (2019). Behavioral, contextual and biological factors associated with obesity during adolescence: A systematic review. PLoS ONE.

[6] Zhou Y., Buck C., Maier W., Von Lengerke T., Walter U., Dreier M. (2020). Built Environment and Childhood Weight Status: A Multi-Level Study Using Population-Based Data in the City of Hannover, Germany. Int. J. Environ. Res. Public Health.

[7] Kim S., Kim G.S. (2019). Ecological Factors Affecting Obesity Among Middle School Students in South Korea. J. Sch. Health.

[8] Ogden C.L., Carroll M.D., Fakhouri T.H., Hales C.M., Fryar C.D., Li X., Freedman D.S. (2018). Prevalence of obesity among youths by household income and education level of head of household—United States 2011–2014. Morb. Mortal. Wkly. Rep..

[9] Chin J.-H., Kim M.-J., Lee J.-H. (2015). Effects of parents BMI, styles and job pattern on the child BMI. Korean J. Growth Dev..

[10] Noh J.-W., Kim Y.-E., Oh I.-H., Kwon Y.D. (2014). Influences of socioeconomic factors on childhood and adolescent overweight by gender in Korea: Cross-sectional analysis of nationally representative sample. BMC Public Health.

[11] Zamora-Kapoor A., Nelson L., Buchwald D. (2016). Maternal correlates of body mass index in American Indian/Alaska Native and White adolescents: Differences between mother/son and mother/daughter pairs. Eat. Behav..

[12] Howe C.J., Alexander G., Stevenson J.L. (2017). Parents’ Underestimations of Child Weight: Implications for Obesity Prevention. J. Pediatr. Nurs..

[13] Lundahl A., Kidwell K.M., Nelson T.D. (2014). Parental Underestimates of Child Weight: A Meta-analysis. Pediatrics.

[14] McKee C., Long L., Southward L.H., Walker B., McCown J. (2016). The role of parental misperception of child’s body weight in childhood obesity. J. Pediatr. Nurs..

[15] Stanton C.A., Green S.L., Fries E.A. (2007). Diet-specific Social Support among Rural Adolescents. J. Nutr. Educ. Behav..

[16] Kim Y., Cardinal B. (2010). Psychosocial correlates of Korean adolescents’ physical activity behavior. J. Exerc. Sci. Fit..

[17] Zarychta K., Mullan B., Luszczynska A. (2016). It doesn’t matter what they say, it matters how they behave: Parental influences and changes in body mass among overweight and obese adolescents. Appetite.

[18] Vepsäläinen H., Nevalainen J., Fogelholm M., Korkalo L., Roos E., Ray C., Erkkola M. (2018). Like parent, like child? Dietary resemblance in families. Int. J. Behav. Nutr. Phys. Act..

[19] Paik J.-A. (2009). The changing nature in the Korean patriarchal family: Continuity and change. Korean J. Humanit. Soc. Sci..

[20] Ruggles S. (2015). Patriarchy, power, and pay: The transformation of American families, 1800–2015. Demography.

[21] Korea Center for Disease Control and Prevention, The Korean Pediatric Society, The Committee for the Development of Growth Standard for Korean Children and Adolescents (2007). Korean Children and Adolescents’ Growth Standard.

[22] Fitzgerald A., Heary C., Kelly C., Nixon E., Shevlin M. (2013). Self-efficacy for healthy eating and peer support for unhealthy eating are associated with adolescents’ food intake patterns. Appetite.

[23] Sallis J.F., Grossman R.M., Pinski R.B., Patterson T.L., Nader P.R. (1987). The development of scales to measure social support for diet and exercise behaviors. Prev. Med..

[24] Roh M.Y., Lee H.K., Lee C.Y., Kim G.S. (2012). Correlates of physical activity among Korean navy personnel: An ecological approach. J. Korean Acad. Community Health Nurs..

[25] Kim Y., Kawachi I. (2016). School- and Individual-level Predictors of Weight Status Misperception among Korean Adolescents: A National Online Survey. PLoS ONE.

[26] Hales C.M., Carroll M.D., Fryar C.D., Ogden C.L. (2017). Prevalence of Obesity Among Adults and Youth: United States, 2015–2016. NCHS Data Brief, No 288.

[27] Seo J.W., Jung J.A., Park H.S., Ko J.S., Kim Y.J., Kim J.Y., Ryoo E., Bae S.H., Sim J.G., Yang H.R. (2008). Assessment of modifiable lifestyle factors for obese children and adolescents through questionnaires. Korean J. Pediatr..

[28] Park H.S., Yim K.S., Cho S.-I. (2004). Gender differences in familial aggregation of obesity-related phenotypes and dietary intake patterns in Korean families. Ann. Epidemiol..

[29] Wan Y., Xu R., Feng H., Zhou Y., Zhang X., Lu L., Tan T., Jiang Y., Chen Z., Wu Y. (2015). Is parental body weight related with their children’s overweight and obesity in Gao Hang Town, Shanghai?. Asia Pac. J. Clin. Nutr..

[30] Pasch L.A., Penilla C., Tschann J.M., Martinez S.M., Deardorff J., De Groat C.L., Gregorich S.E., Flores E., Butte N.F., Greenspan L.C. (2016). Preferred Child Body Size and Parental Underestimation of Child Weight in Mexican-American Families. Matern. Child Health J..

[31] Jain A., Sherman S.N., Chamberlin L.A., Carter Y., Powers S.W., Whitaker R.C. (2001). Why don’t low-income mothers worry about their preschoolers being overweight?. Pediatrics.

[32] Birch L., Ventura A. (2009). Preventing childhood obesity: What works?. Int. J. Obes..

[33] Berge J.M., Wall M., Bauer K.W., Neumark-Sztainer D. (2010). Parenting characteristics in the home environment and adolescent overweight: A latent class analysis. Obesity.

[34] Temple J.L., Wrotniak B.H., A Paluch R., Roemmich J.N., Epstein L.H. (2006). Relationship between sex of parent and child on weight loss and maintenance in a family-based obesity treatment program. Int. J. Obes..

[35] Erkelenz N., Kobel S., Kettner S., Drenowatz C., Steinacker J.M. (2014). Parental activity as influence on children’s BMI percentiles and physical activity. J. Sports Sci. Med..

[36] Keresztes N., Piko B.F., Pluhar Z.F., Page R.M. (2008). Social influences in sports activity among adolescents. J. R. Soc. Promot. Health.

[37] Gustafson S.L., Rhodes R.E. (2006). Parental correlates of physical activity in children and early adolescents. Sports Med..

[38] Ministry of Health and Welfare (2015). The 4th National Health Plan (2016–2020).

